# Experimental Investigation and FEM Simulation of the Tensile Behavior of Hot-Rolled Quenching and Partitioning 5Mn Steel

**DOI:** 10.3390/ma18040868

**Published:** 2025-02-17

**Authors:** Firew Tullu Kassaye, Tamiru Hailu Kori, Aleksandra Kozłowska, Adam Grajcar

**Affiliations:** Department of Engineering Materials and Biomaterials, Faculty of Mechanical Engineering, Silesian University of Technology, 18A Konarskiego Street, 44-100 Gliwice, Poland; firew.tullu.kassaye@polsl.pl (F.T.K.); tamiru.hailu.kori@polsl.pl (T.H.K.); aleksandra.kozlowska@polsl.pl (A.K.)

**Keywords:** tensile behavior, mechanical properties, finite element simulation, quenching and partitioning, hot rolling, medium Mn steel

## Abstract

Medium manganese steels provide a good combination of tensile strength and ductility due to their multiphase microstructure produced during the multi-step heat treatment process. This study primarily focused on testing and analyzing the tensile properties of 0.17C-5Mn-0.76Al-0.9Si-Nb medium manganese quenching and partitioning (QP) steel using both the experimental and finite element method (FEM) in the multilinear isotropic hardening material model. The 7 mm and 12 mm thick plates exhibited a similar microstructure of tempered primary martensite, lath-type retained austenite, and secondary martensite. The experiments measured tensile strengths of 1400 MPa for 12 mm round specimens and 1325 MPa for 7 mm flat specimens, with total elongations of 15% for round specimens and 11% for flat specimens. The results indicated that the sample’s geometry has some effect on the UTS and ductility of the studied medium-Mn QP steel. However, the more important is the complex relationship between the plate thickness and yield stress and ductility, which are affected by finishing hot rolling conditions. The FEM results showed that the von Mises stresses for flat and round specimens were 1496 MPa and 1514 MPa, respectively, and were consistent with the calculated true stresses of experimental results. This shows that numerical modeling, specifically a multilinear isotropic hardening material model, properly describes the material properties beyond the yield stress and accurately predicts the plastic deformation of the investigated multiphase QP steel.

## 1. Introduction

First generation advanced high-strength steels (AHSS), such as dual-phase (DP) and transformation-induced plasticity (TRIP) steels, offer excellent mechanical properties for automotive applications [1,2,3,4,5]. The mechanical properties of DP steels are greatly influenced by microstructural features, such as the martensite volume fraction, ferrite grain size, and martensite morphology [6]. The mechanical properties of TRIP steels are controlled to a considerable extent by the volume fraction, carbon content, and distribution of retained austenite [7,8]. Second generation AHSS, including high-manganese twinning-induced plasticity (TWIP) steels, have excellent mechanical properties due to their TWIP mechanism, influenced by stacking fault energy (dependent on a chemical composition and temperature), grain size, and deformation conditions [9,10,11,12]. Third generation AHSS steels, containing a medium manganese content (3–12 wt.%), include retained austenite (RA), which enhances strain hardening through TRIP or TWIP effects. The most important steels that have this behavior are medium manganese steels (medium-Mn), quenching and partitioning (Q&P) steels, and carbide-free bainitic (CFB) steels [13,14].

Medium-Mn steels are developing as a promising category of AHSS, with exceptional mechanical qualities and potential for automotive applications [15,16,17]. These steels are known for their high strength, excellent ductility, and low yield ratio, making them suitable for easy metal-forming operations. They can be modified through various processing methods like quenching, partitioning, warm rolling, and intercritical annealing [18,19]. Grajcar et al. [20] found that medium-manganese steel with 3 Mn wt.% had interlath and blocky-type RA. The stability and transformation of RA into strain-induced martensite during deformation significantly influence their mechanical properties [21]. Recent research on medium-Mn steels has been aimed at enhancing the material properties for automotive or similar applications. In these steels, RA transforms via the strain-induced martensitic transformation (SIMT) during metal forming, which influences strength and formability [22].

Among the most promising candidates for industry applications, Q&P steels exhibit significantly higher strength compared to TRIP and DP steels, while also requiring fewer alloying element additions compared to TWIP steels. Since the first report by Speer et al. in 2003 [23], Q&P steels have drawn significant attention because of their high strength and improved ductility (with respect to the first generation AHSSs). The Q&P process makes steels stronger and more flexible by tailoring the amount of lath-type RA. This treatment is applicable to all types of steels, including martensitic stainless steel [24]. Liu et al. [25] studied the influence of strain rate on the tensile deformation behavior of Q&P steel and observed that higher strain rates enhanced steel strength. Choi et al. [26] used microstructure-based finite element modelling to study the impacts of constituent properties on Q&P steel performance, finding that higher austenite stability and smaller strength variation between phases typically enhanced tensile properties performance. In contrast to typical cold-rolled and annealed DP or TRIP steels, Q&P steels are usually manufactured by hot rolling and controlled cooling [27], which makes them suitable for structural applications requiring higher plate thicknesses. Other studies [28,29] primarily focused on sheet products of Q&P medium-Mn steels, with limited research on plate products. This study offers alternative materials for achieving a combination of high strength and reasonable ductility in medium-manganese Q&P steels on plate products. The relationship between plate thickness, specimen geometry, tensile strength, and ductility, which are affected by finishing hot rolling conditions, has not been extensively investigated in the context of advanced high-strength Q&P steels, which is the focus of the current study.

Experimental tensile tests offer crucial insights into mechanical properties, including Young’s modulus, ultimate tensile strength, and stress-strain relationships. The effect of specimen geometry on the mechanical properties of steel has been extensively investigated. Calik et al. [30] have shown that for both boron and boron-free AISI 1040 steels, the boride layer thickness and hardness are independent of the quality of the structure, while the ultimate tensile strength and elongation are affected by the specimen geometry. Similarly, for sheets and plates, the specimen width and thickness affect the stress-strain relationship [31].

Numerical simulations provide valuable information on tensile behavior across different materials and testing methods. The study [32] used the ANSYS software to conduct numerical tensile tests on three types of steel sheets, examining the impact of specimen geometries and mesh sizes on stress-strain curves, which is crucial for understanding material behavior under loading. Gholipour et al. [33] used the Gurson–Tvergaard–Needleman (GTN) damage model to look into how ductile fracture happens in SAE 1010 steel. The results of the finite element simulations were relatively accurate, and the experimental data showed how steel fails when it comes to ductile fracture under shear and tension forces. They also suggested a generalized simulation method that can be used to obtain a better idea of the same phenomenon for both mild and high-strength steel [34]. Wang et al. [35] used an experimental numerical combined method to find the true stress-strain relationship of tensile specimens after necking without using finite element analysis (FEA). They achieved this by minimizing the discrepancy in error between the load displacement curves from the experiment and the FEA. Numerical simulation is a faster and safer alternative to physical testing, allowing researchers to effectively examine material characteristics and optimize design processes [36]. Numerical simulation is used to overcome the difficulties caused by necking phenomena and to obtain the true stress-strain curves for both round and flat steel specimens [37].

In this study, the effect of plate thickness and specimen geometry (size and shape) on the tensile behavior of hot-rolled 0.17C-5.0Mn-0.76Al-0.9Si-0.06Nb multiphase steel was carried out. This study employed experimental investigation and FEA to analyze the characteristics of the tensile behavior of newly developed medium-Mn Q&P steels. The combined methods enhance reliability and demonstrate potential for material design and performance optimization. There are no comprehensive studies on this issue that utilize both experimental and numerical methods to investigate the potential of FEA to predict the effect of specimen geometry on the tensile properties of medium-Mn Q&P steels. This study will fill this gap and expand the knowledge in AHSS beyond optimizing product performance and their applications.

## 2. Materials and Methods

### 2.1. Materials

The Q&P steel used in this study has a medium manganese content with a chemical composition (wt.%) of 0.17C, 5.0Mn, 0.76Al, 0.88Si, and 0.063Nb (Table 1). Q&P is a heat treatment process that produces a microstructure characterized by a martensitic matrix and some amount of RA. The partial quenching regulates the martensite-austenite ratio and partitioning, thereby stabilizing the austenite at room temperature. 

**Figure 1:**
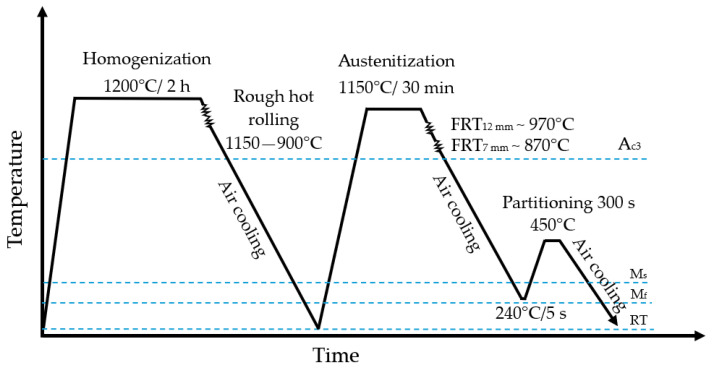
Processing schedule of investigated steel (FRT—finishing rolling temperature).

The steel under investigation was cast using a vacuum induction furnace. Following casting, the steel underwent austenitization at 1200 °C for a duration of 2 h and was subsequently roughly hot-rolled within a temperature range of 1150–900 °C to achieve a thickness of 22 mm. Subsequently, the steel was reheated to 1150 °C for a duration of 30 min. The material was subsequently hot rolled in 3 or 5 passes, achieving final thicknesses of 12 mm and 7 mm, respectively. The final pass occurred at a temperature of 970 (12 mm plate) or 870 °C (7 mm plate). The time-temperature variants of QP treatment were studied at quenching temperatures in a range of 240–300 °C and for the different partitioning temperatures in a range of 350–450 °C. The partitioning time in a range of 90–1800 s was also investigated. The results obtained showed that a quenching temperature of 240 °C and isothermal holding at partitioning temperatures between 400 °C and 450 °C for 300–900 s are the most beneficial in terms of stabilizing a significant fraction of RA and minimizing the fraction of undesired secondary martensite and bainite in the microstructure. Therefore, the quenching temperature of 240 °C and the partitioning temperature of 450 °C (300 s) variants were selected for this study. Following the final pass, plates were air-cooled to the quenching temperature of 240 °C to obtain the microstructure composed of about 85% of martensite and 15% of austenite. Then, the plates were subsequently placed in an electric furnace maintained at 450 °C for a duration of 300 s to provide carbon partitioning from C-rich martensite into austenite to stabilize this phase at room temperature. The hot rolling process was conducted utilizing a LPS/B semi-industrial line at the Łukasiewicz Research Network, Upper Silesian Institute of Technology in Gliwice, Poland [38]. Figure 1 illustrates the processing schedule of the examined steel.

### 2.2. Experimental Procedure

**Figure 2:**
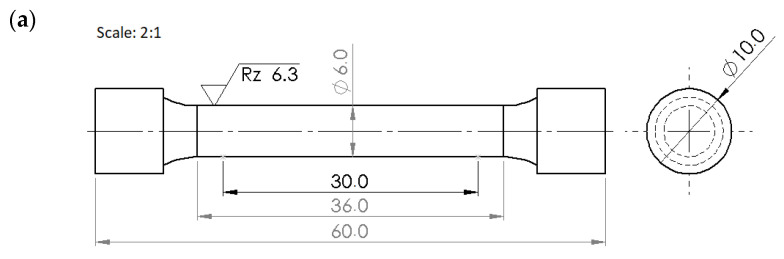

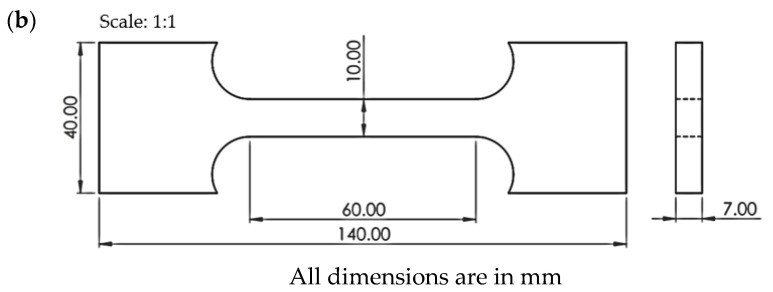
Tensile test specimen dimensions: (**a**) round specimen from 12 mm thickness plate; (**b**) flat specimen from 7 mm thickness plate.

The cylindrical and flat tensile test specimens were machined from hot-rolled plate material, aligning the tensile test axis with the plate rolling direction. These cylindrical specimens were machined from 12 mm plate thickness, measuring a gauge length $\;(L_{O})$ of 30 mm and a diameter of 6 mm for the circular cross-section ($A_{0}$) (Figure 2a). The prepared flat specimens from 7 mm plate thickness had 10 mm in width, 60 mm gauge length, and 7 mm in thickness, as illustrated in Figure 2b. The calculated slimness ratio ($L_{O}⁄\surd (A_{0})$) was 5.65 and 7.13, respectively, for round and flat samples. To remove the layers developed through machining, the gauge areas of the sections were initially grinded with metallographic sandpaper and further polished with 0.5 μm diamond paste. The ZWICK universal testing machine (Zwick Roell, Ulm, Germany) was used to conduct tensile tests at room temperature at a strain rate of 0.001 s^−1^ on three samples of each variant to obtain the averaged mechanical properties. All tests met PN-EN ISO 6892-1: 2020-05 standards [39].

The specimens were subjected to a uniaxial tensile load along their axis during the tension test, which caused deformation until their failure. The output of the tensile test is displayed as force versus elongation. These force versus elongation curve values measure mechanical properties such as ultimate tensile strength, yield strength, Young’s modulus, total elongation, and uniform elongation. Engineering stress and strain are defined by Equations (1) and (2), respectively.

(1) $$\sigma \;=F/A_{0}$$

(2) $$\varepsilon =∆L/L_{O}$$

where σ is the engineering stress, ε is engineering strain, F is the applied force, $A_{0}$ is the original cross sectional area, ∆L is a change in length, and Lo is an original length before the applied load.

True stress-strain values derived from tensile tests are the details of the material response to loads, which are not provided in the engineering stress-strain. True stress and strain are not based on the initial dimensions. Instead, they show how the cross-sectional area and length change as the material deforms. This gives a more accurate behavior of how the material will behave until it breaks [40]. True stress and strain values were calculated from engineering stress-strain data and defined by Equations (3) and (4):

(3) $$\sigma_{T}=\sigma \;\left(1+\varepsilon \right)$$

(4) $$\varepsilon_{T}=\ln\left(1+\varepsilon \right)$$

where $\sigma_{T}\;is\;the\;true\;stress,\;\varepsilon_{T}$ is the true strain, and *σ* and *ε* are engineering stress and strain, respectively.

### 2.3. Microstructural Characterization

To characterize microstructural details, the samples after the Q&P heat treatment (before tensile tests) were observed using a Zeiss Supra 25 scanning electron microscope (SEM) (Carl Zeiss AG, Jena, Germany) working in a secondary electron (SE) mode. The samples for microstructural investigations were prepared by mechanical grinding with increasingly finer abrasive SiC papers (up to 2000 grid). Then, they were polished with a diamond paste of particle sizes 3 and 1 μm and finally etched in 3% nital solution for 5 s to reveal the microstructure.

The volume fraction of RA was estimated based on the X-ray diffraction (XRD) analysis using the Rietveld refinement method. The volume fraction of RA was determined based on the average results of three measurements. The Panalytical X’Pert Pro MPD diffractometer (PANalytical, Almelo, The Netherlands) equipped with a cobalt anode working in Bragg–Brentano geometry at 40 kV and 30 mA was used for the diffraction analysis. The scans were collected in the θ–2θ range with a step size of 0.01°. The identification of the phases was based on the data contained in the International Center for Diffraction Data (ICDD) database.

### 2.4. Finite Element Simulation

In this study, ANSYS version 2021R1 software was used for finite element simulation of the tensile test. The geometry of the tensile specimen was defined by considering standard dimensions (Figure 2a,b). A 3D model was created using the Solid Works 2024 version and saved in the IGS format. This file was then imported into ANSYS, and a face split was conducted using the ANSYS design modeler to control mesh and FEA using the ANSYS workbench. Material properties in the numerical model were assigned based on experimental data from actually investigated steel samples. The model captures the material property beyond the yield point, using a multilinear isotropic hardening material model to predict plastic deformation accurately. A multi-linear isotropic hardening model is chosen for this study for greater accuracy in correlated ultimate tensile strength and uniform elongation with experimental data [41]. Isotropic hardening, a parameter in plasticity theories, offers a reasonable approximation for monotonic loading. However, it is dependent upon plastic strain and requires experimental determination. Multilinear isotropic hardening laws are used in FEM models to analyze plastic behavior, focusing on the yield surface’s centered expansion with plastic strain development, and it represents the material’s stress-strain behavior through a series of defined points [42]. When compared to other models, the multilinear isotropic hardening model captures essential hardening characteristics and balances accuracy and computational efficiency, while elastic-perfectly plastic models are simpler but fail to capture strain hardening, crucial for high-strength steels [43]. The kinematic hardening model accounts for the Bauschinger effect but requires additional parameters and is more suited for cyclic loading [44]. Combined isotropic-kinematic hardening models offer a comprehensive approach but are more complex to implement and may not be necessary for this study, which focuses on monotonic loading cases.

**Figure 3:**
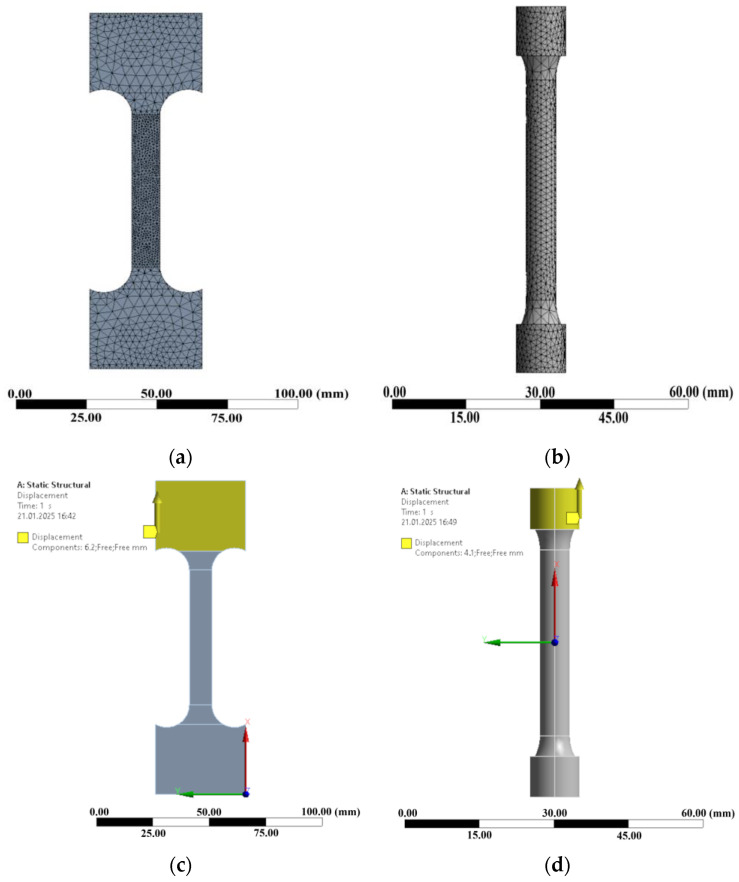
Meshing: (**a**) round specimen; (**b**) flat specimen; boundary condition: (**c**) flat specimen; (**d**) round specimen: the round specimen from 12 mm thickness plate; the flat specimen from 7 mm thickness plate.

In elastic and elasto-plastic analyses, linear tetrahedral elements outperform with superior accuracy in displacement, stress, and plastic flow calculations [41,42]. Mesh convergence studies were performed on the specimen using linear tetrahedral elements with mesh sizes of 3 mm, 2.5 mm, 2 mm, 1.5 mm, 1 mm, and 0.5 mm. Mesh size of 0.5 mm with linear tetrahedral elements showed stable values of strain, stress, and displacement. Thus, a mesh size of 0.5 mm was set in the gauge and stress concentration regions to provide an optimal distribution of stress and strain, and 1.5 mm was used in other regions of the specimen to ensure computational efficiency (Figure 3a,b). Boundary conditions were applied by fixing one end of the specimen, while the other end was set with a displacement boundary condition to simulate tensile loading by incrementally pulling the specimen in the axial direction, as shown in Figure 3c,d. The true stress-strain data were used as input data to simulate the tensile behavior of the materials based on local stress and strain [45]. The average true stress and strain was calculated from average engineering stress and strain tensile tests using Equations (3) and (4). To obtain multi-linear hardening curves, plastic strain and true stress were applied to the specimen as tabulated data in the ANSYS 2021R1 model. 

## 3. Results

### 3.1. Microstructure

**Figure 4:**
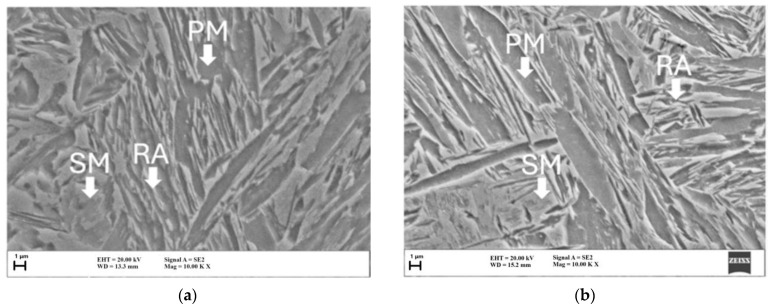
SEM micrographs after quenching at 240 °C and partitioning at 450 °C (300 s): (**a**) plate 7 mm; (**b**) plate 12 mm. PM—primary martensite; SM—secondary martensite; RA—retained austenite.

Microstructures of investigated plates with a thickness of 7 and 12 mm after quenching and partitioning are presented in Figure 4. To precisely determine the volume fraction of RA after quenching at 240 °C and partitioning at 450 °C for 300 s, X-ray diffraction measurements were performed. The fractions of RA estimated for 12mm and 7mm plates were 14.5 vol.% and 12.1 vol.%, respectively. Both plates consist of microstructures of primary martensite (PM), which was formed during quenching and was tempered during the partitioning step, and some fraction of lath-type RA, which was retained in the microstructure after quenching and stabilized during the partitioning step. Moreover, some fraction of secondary martensite (SM) was also formed in both microstructures during the final cooling of austenite, showing low stability of less C-enriched areas (higher local M_s_ temperature).

The carbon diffusion from martensite into austenite during a partitioning step has a critical effect on the microstructure evolution of Q&P steels. The partitioning process significantly influences the stability of RA, which is a phase that plays a key role in enhancing the ductility of TRIP-aided steels. The driving force for diffusion from supersaturated martensite into RA is the chemical gradient caused by the difference in carbon content between these microstructural constituents. The intensity of C diffusion depends on time-temperature parameters of the partitioning step. Higher temperatures accelerate the intensity of diffusion; however, it may lead to carbide precipitation and therefore less carbon is available in solid solution to stabilize RA. The austenite with low stability easily transforms into martensite during final cooling or at a small strain, leading to reduced work hardening rate of steel. The over-stabilized RA may not transform into strain-induced martensite. This diminishes the efficiency of the TRIP effect, limiting the strain hardening and ductility of steel [46,47].

### 3.2. Experimental Tensile Tests

**Figure 5:**
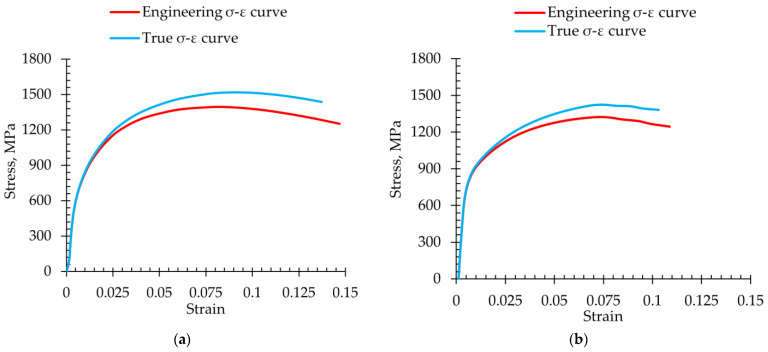
Comparison of averaged engineering and true stress-strain curves of investigated Q240P450(300 s) steel: (**a**) 12 mm thickness round specimen; (**b**) 7 mm thickness flat specimen.

The average engineering curves and the true stress-strain curves of 12 mm and 7 mm plates of investigated steel are presented in Figure 5. The results show some differences between the curves registered for different plate thicknesses. Engineering curves do not fully reflect the material’s behavior under high deformation due to necking formation, which distorts the stress-strain response [48]. The true stress-true strain curves take the reduced area into account.

From the experimental results, the microstructural and mechanical properties of the 7 and 12 mm thick plates of investigated steel provide details on the effect of microstructure and plate geometry on tensile properties. The two plates showed identical microstructural compositions, which included PM, lath-type RA, and SM. Despite the homogeneity of microstructural features, the two plate thicknesses differed in yield strength (YS) and ultimate tensile strength (UTS) and significantly in total elongation (TEl). The 12 mm thick plate had UTS of 1400 MPa, corresponding to a true stress of 1514 MPa and a TEl of around 15%, corresponding to a true strain of 0.137. Both values exceeded the 7 mm thick plate, which presented UTS of 1325 MPa and TEl of around 12% with corresponding true yield stress $\;(\sigma_{T\;YS0.2})$, true ultimate tensile stress $\;{(\sigma }_{T\;UTS})$, true total elongation strain ($\varepsilon_{T\;TEl})$, and true uniform elongation strain ($\varepsilon_{T\;UEl}$), as summarized in Table 2.

**Figure 6:**
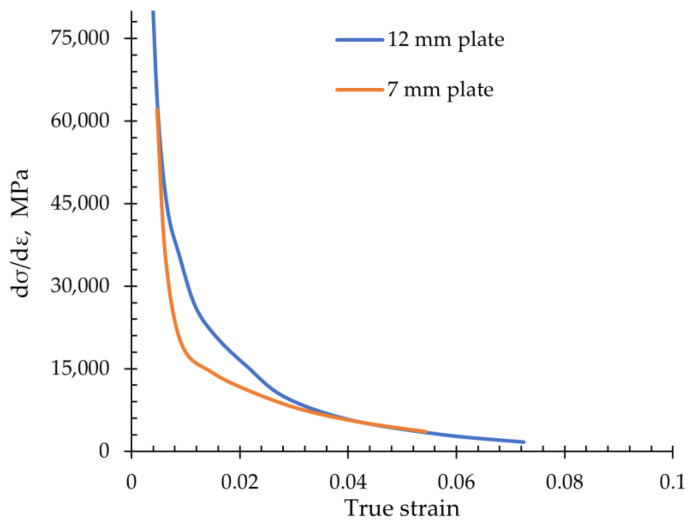
Strain hardening rate curves as a function of true strain for both plate thickness.

The relationship between strain hardening rate (dσ/dε) and true strain for 12 mm and 7 mm thick plates, showing the difference in their work hardening opportunities, is presented in Figure 6. Initially, as the strain increases, the strain hardening rate of the two plates decreases, but this occurs more rapidly for the more strengthened 7 mm plate. However, for both specimens, there is a clear change at the true strain values of approximately 0.01 and 0.02, where dσ/dε slowly decreases during deformation, indicating some potential for further work strengthening. After this, the strain hardening rate starts to decrease continuously. This is related to the phase transition from RA to strain-induced martensite [49,50,51].

### 3.3. FEM Simulation Tensile Test

**Figure 7:**
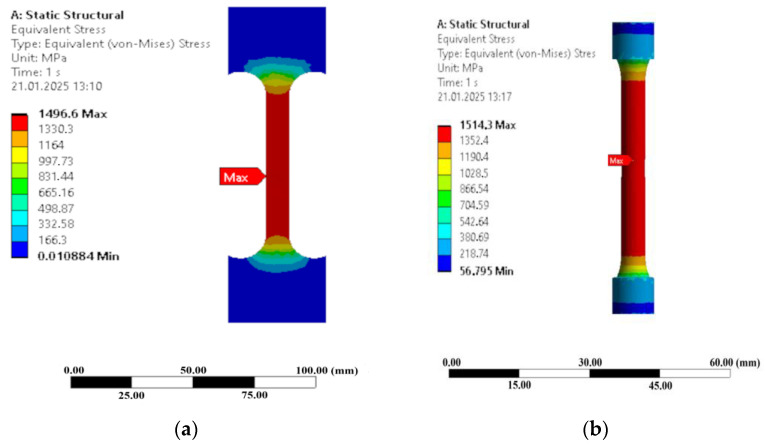
FEM simulation results of equivalent (von-Mises) stresses for Q240P450(300 s): (**a**) flat specimen; (**b**) round specimen.

A study on the influence of mesh size on ultimate tensile strength (UTS) in round and flat specimens was conducted using linear tetrahedral elements for mesh sensitivity analysis. Results showed that decreasing mesh size led to a slight increase in UTS for round specimens, with UTS values reaching 1500 MPa for 3 mm mesh size, 1502 MPa for 2 mm, 1512 MPa for 1 mm, and 1514 MPa for 0.5 mm. However, flat specimens showed minimal variation in UTS with mesh refinement. The stress distribution in the finite element simulation results are shown in Figure 7. Both the flat and round specimens exhibited high stress concentration in the center of the gauge section, where von-Mises stresses were 1496 MPa and 1514 MPa, respectively, with linear tetrahedral elements of 0.5 mm mesh size. The findings indicated that the FEM was consistent with the experimental data, hence validating the simulation’s accuracy as well.

## 4. Discussion

### 4.1. Effect of Q&P Processing on the Microstructure and Mechanical Properties

The microstructural analysis indicates that PM, produced during quenching and tempered during partitioning, represents the primary strengthening phase [52], whereas RA, stabilized during partitioning, improves ductility via the TRIP effect [53]. The higher RA fraction, the round sample’s geometry, and a higher finishing rolling temperature contributed to obtaining a higher elongation of the 12mm plate (Table 2). The work hardening rate of 7mm sample was higher when compared to the 12mm plate and thus higher YS = 809 MPa was noted. The formation of SM during final cooling from low-stable austenite introduces additional martensitic phases, which may improve overall hardness and strength [54]. What is positive is that the plate thickness did not significantly affect the proportions or morphology of the microstructural constituents, which indicates that the heat treatment process effectively transformed the microstructure uniformly through the cross section of both specimens.

The yield strengths observed in the present study were 809 MPa (7 mm plate) and 684 MPa (12 mm plate). The UTS values were 1423 MPa (7 mm plate) and 1514 MPa (12 mm plate). Typically, the Q&P heat treatment process is performed on sheets with a thickness not exceeding 4 mm [29,55]. Ensuring a uniform microstructure across the cross-section and resulting mechanical properties of plates is challenging due to the cooling rate gradient across their cross-section [56]. Somani et al. [57] and Zhou et al. [58] investigated the microstructure and mechanical properties of Q&P plates containing 0.7–2.0 wt.% Mn with a thickness up to 20 mm. They observed inhomogeneous microstructure at the cross-section of plates, affecting their hardness. The martensite was observed at the surface, and the microstructure composed of martensite and bainite was formed in the core. In our study, microstructures at the cross section of both 7-mm and 12-mm plates were homogenous due to the high hardenability of steel provided by the addition of 5 wt.% of Mn.

Typically, medium-Mn steels with a martensitic matrix show higher strength properties than ferritic or bainitic medium-Mn steels [59,60]. The high yield strength in a range of 600–1000 MPa and tensile strength of 1200–1600 MPa can be achieved in medium-Mn Q&P steels mainly through strengthening contributions from martensite and the tailored stability of RA. Kaar et al. [29] investigated the mechanical properties of medium-Mn Q&P sheet steel containing ~4.5 wt.% Mn and Si/Al addition. They noted that YS was in a range of 700–1000 MPa and UTS of 1100–1600 MPa. Similar strength properties were observed in our study. Cai et al. [55] investigated the tensile properties of a cold-rolled Fe-0.2C-6Mn-1Al Q&P steel. They observed YS and UTS at a level of about 900 and 1500 MPa, respectively. The slightly higher strength properties when compared to the results of our study are mainly related to the high Mn content and cold deformation process.

### 4.2. Influence of Plate Thickness and Specimen Geometry on the Mechanical Properties

The study examined the impact of microstructure and plate geometry on tensile properties of 7 and 12 mm thick plates. Even though they had identical microstructural compositions, the thicknesses differed in yield strength, ultimate tensile strength, and total elongation. The 12 mm thick plate had higher UTS and TEl values. Variations are due to the interplay between the specimen geometry and plate thickness. The sample geometry plays a crucial role in determining material mechanical behavior. It influences tensile properties (tensile strength and elongation) [31,32], stress distribution, and strain localization [30], which are essential for optimizing high-strength materials performance. The 7 mm flat sample had a slimness ratio of 7.13, which is higher compared to the normative slimness ratio of 5.65 for the round sample machined from the 12 mm plate. From the literature [61], it is clear that the higher the value of the slimness ratio, the lower the ductility, because the larger volume of material transfers stress more effectively and improves uniformity, minimizing early localization and necking. This indicates that when the slimness ratio increases, the percentage of elongation typically decreases. High slimness ratio specimens are more likely to experience necking within the gauge length, allowing testing equipment to capture the stress-strain response until fracture. Conversely, specimens with low slimness ratios may experience necking or failure outside the gauge length or near stress concentration zones [62]. In addition, high slimness ratio experiences increased stress concentration at specific points, particularly at imperfections, leading to localized areas of high stress, potentially causing early failure [63]. Moreover, while the microstructural constituents are identical, the 12 mm thick plate, assessed as a round specimen, experiences more homogenous stress distribution and less strain localization during tensile deformation [48]. In contrast, the thinner 7 mm plate, tested as a flat specimen, exhibited stress concentration and was more prone to strain localization and edge effects, resulting in premature necking and reduced elongation. Specimens with round geometry show better UTS and ductility due to the round specimens’ symmetrical circular cross-section area and a more homogeneous distribution of stresses, which may delay necking. When deformation concentrates in a small area, strain localization leads to failure [64,65]. This homogeneity reduces the probability of stress concentrations, which can lead to rapid localized yielding. The thicker plate enabled more consistent stress distribution and strain hardening, leading to enhanced strength and increased ductility. This indicates a clear correlation between the ductility and specimen geometry [66].

The round specimen from the 12 mm thickness plate showed higher total elongation, indicating that the thickness and geometry of the specimens influence the stress distribution near the necking region. Thick specimens stretch more after necking, which strengthens the link between the specimen geometry and tensile properties [67,68]. On the other hand, sharp corners in flat samples cause stress concentration, increasing localized stress compared to round specimens. The round specimens have a more uniform strain distribution during tensile testing compared to flat specimens [69,70]. This is because flat shape specimens experience non-uniform stress distribution under tensile loading conditions, leading to increasing strain localization in shear bands or deformation zones at edges.

The highest difference between two plate thicknesses was registered for yield stress values, which were 683 MPa and 808 MPa, with corresponding true stress values listed in Table 2. Since the microstructure is the same and homogeneous through the cross section of both plates, and from the literature it is known [61,71] that the sample geometry does not affect YS, this difference must be related to other factors. The only explanation is the different finishing rolling conditions. The finishing rolling temperature was 970 °C for a 12 mm plate and 870 °C for a 7 mm plate, which defines different work strengthening of the austenite subjected to controlled cooling. The lower FRT generates more dislocations in the material due to less advanced static recrystallization and recovery processes [72], revealing pancaked austenite grains in the microstructure. This is a reason for significantly higher YS for the 7 mm plate with the FRT ~870 °C. The higher dislocation density in RA inherited from the austenite deformed at 870 °C affects the strain-induced martensitic transformation proceeding during tensile loads. This effect, together with general higher microstructure hardening and limited opportunity for further work strengthening (corresponding to a more rapid decrease of work hardening rate in Figure 6), means that the UTS of a 7 mm plate is a little bit smaller. The consequence is also the lower UEl value for this plate. As discussed earlier, the smaller TEl of the 7 mm plate is related to the geometry of the sample and a higher value of slimness ratio = 7.13.

The differences in FRT are reflected in different yield strength results and in hardness measurements as well. The hardness difference between two finishing rolling temperatures was ~15 HV1, with the higher value of 408 for the thinner 7 mm plate, which is in accordance with the yield strength tendency. The direct microstructural evidence of the FRT effect on the mechanical behavior, hardness, and dislocation density is the aim of the ongoing study. The finishing rolling temperature affects the recrystallization kinetics in austenite and the density of dislocations. As the rolling temperature decreases, the dislocation mobility decreases, and the effectiveness of the recovery and recrystallization is limited. Liu et al. [73] observed significant refinement of austenite grains and further formation of finer martensite laths in Q&P steel when the finishing rolling temperature was reduced from 920 to 840 °C. They also noted that reducing the finishing rolling temperature to 840 °C improved the stability of RA and allowed for an increase in yield strength, ultimate tensile strength, and elongation.

The relationship between strain hardening rate (dσ/dε) and true strain for 12 mm and 7 mm thick plates, showing the difference in their work hardening opportunities, is presented in Figure 6. Initially, as the strain increases, the strain hardening rate of the two plates decreases, but more rapidly for the more strengthened 7 mm plate. However, for both specimens, there is a clear change at the true strain values of approximately 0.01 and 0.02, where dσ/dε slowly decreases during deformation, indicating some potential for further work strengthening. After this, the strain hardening rate starts to decrease continuously. This is related to the phase transition from RA to strain-induced martensite [49,50,51].

Results obtained in the present study show that the Q&P heat treatment can be applied to high-strength medium-Mn steel plates with increased thickness, which can be used for critical construction elements of trucks and cars [56]. QP steels exhibiting tensile strengths ranging from 1200 to 1500 MPa are appropriate for high strength applications, such as automobile structural components and forged parts, while elongation values between 10–15% are ideal for static load-bearing applications [4].

The present study encompassed a limited range of plate thicknesses, strain rates, quenching and partitioning temperatures, and partitioning times. Future studies should further explore these parameters to understand their impact on mechanical properties. The variations in partitioning temperatures can affect the tensile strength and ductility of a material due to the distinct fractions of retained austenite and secondary martensite [74]. The partitioning time also affects the stabilization of retained austenite and carbide formation [75] and microstructural development [76]. It was reported in the literature [77,78,79] that strain rate impacts the mechanical properties of medium-Mn steels, affecting the TRIP effect’s. An increase in strain rate increases RA mechanical stability, reducing TRIP effectiveness and affecting work hardening rate, tensile strength, and elongation. An increase in mechanical stability of RA is related to an increase in its stacking fault energy (SFE) [80]. This study focuses on material behavior at a single strain rate (0.001 s^−1^), but future studies will further consider the effect of different strain rates on plate thicknesses and specimen geometry.

### 4.3. The Comparison of Experimental Results and FEM Simulation

**Figure 8:**
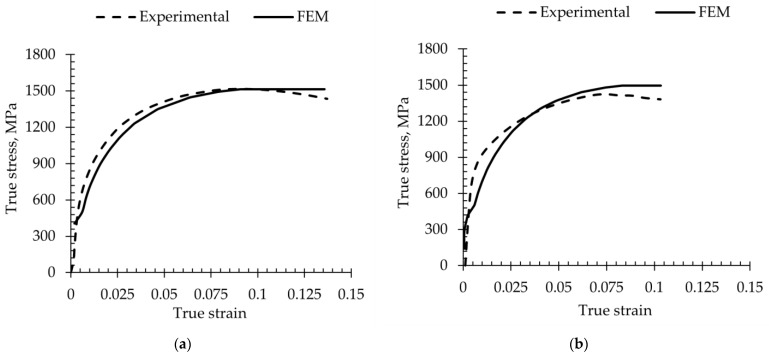
Comparison of experimental and FEM stress-strain curves: (**a**) Q240P450(300 s)—12 mm plate thickness; (**b**) Q240P450(300 s)—7 mm plate thickness.

True stress strain has been plotted to show the difference between experimental and FEM values. Figure 8 compares the experimental and FEM stress-strain curves. The FEM simulation corresponds well with the experimental data, indicating that the multilinear model adequately captured the studied steel response under plastic behavior conditions. This correlation continues beyond the yield point and into early plastic deformation up to a point where the multilinear model demonstrates its capability in simulating strain hardening. However, at the ultimate stress, a small deviation exists. This variation may be attributed to the inability of the multilinear model to fully account for microstructural changes and damage initiation with increasing strain. The FEM stress-strain curve validates the use of a multilinear material model in the FEM simulation to more accurately represent the complex plastic behavior of the investigated multiphase steel, a finding that is consistent with other studies [81,82].

As the strain increases, the FEM stress-strain curve increases simultaneously with the experimental data, indicating that the model can capture the strain hardening. Beyond the ultimate stress, the experimental curve exhibited softening and then decreased, indicating necking and material failure progression. In contrast, the FEM simulation, constrained by the multilinear model, maintains the ultimate stress over a wider strain range and demonstrates a limited ability to predict the softening behavior. The experimental results and predictions from FEM simulations were compared, and the percentage error was calculated using Equation (5), ensuring consistency and accuracy in measuring differences.

(5) $$Error\left(\%\right)=\left(\left|\frac{FEA\;result-Experimental\;result}{Experimental\;result}\right|\right)\times 100$$

Table 3 compares experimental results and FEM predictions for flat and round specimens of various thicknesses. The true stress corresponding to UTS for the flat specimen was slightly lower than the one predicted by FEM, with a small error of 5%. This suggests good agreement between experimental and FEM values for ultimate tensile strength. Experimental testing showed that the thick specimen had greater uniform strain (0.097) compared to the 7 mm plate specimen (0.074), indicating the influence of sample geometry on ductility. The experimental and FEM results for elongation in both round and flat specimens showed good agreement, with percentage errors of 12%. The study concluded that the FEM multilinear material model accurately predicted the UTS and UEl values of medium-Mn QP steel in both flat and round specimens, with a slight difference between experimental values The comparison results also show that FEM multi-linear isotropic hardening matched the experimental results for ultimate tensile strength and uniform elongation [41]. However, it has limitations in accurately capturing total elongation beyond the necking point. In Table 3, the incomplete total strain elongation data of FEM represents the limitation of multilinear material modeling in capturing total deformation at fracture points when compared to experimental results. Future research should combine multilinear modeling with advanced ductile fracture models to address these limitations.

## 5. Conclusions

The microstructural study of hot rolled 5Mn steel plates of different thicknesses (7 mm and 12 mm) revealed a homogeneous phase composition, which included primary martensite for strength, retained austenite for ductility via the TRIP effect, and secondary martensite for enhanced hardness. The results show that the investigated plate thicknesses did not significantly influence the microstructural morphology, indicating a homogeneous microstructure after the multi-step QP heat treatment. The results also illustrate the efficiency of the quenching and partitioning process in attaining a good balance of strength and ductility across various plate thicknesses.

According to the experimental results, plate thickness and specimen geometry affect the ultimate tensile strength slightly but significantly influence the ductility of the investigated multiphase QP steel. The flat samples with a slimness ratio of 7.13 are characterized by the lower UTS = 1325 MPa and TEl = 11.1%, whereas the round samples with a normative slimness ratio of 5.65 made of 12 mm plate show the best values of UTS = 1400 MPa and TEl = 15.2%. The highest difference between the two plates was registered for yield stress equal to 808 MPa and 683 MPa, respectively, for 7 mm and 12 mm plates. However, the homogenous microstructure of both plates indicates that this is mainly attributed to different finishing rolling temperatures, which were 100 °C lower for the 7 mm plate. The higher dislocation density and overall microstructural strengthening in this plate is manifested by its higher YS and lower ductility.

The FEM stress-strain curves have good agreement with the experimental curves. The results show that the FEM model accurately predicts the work strengthening behavior and plastic deformation of a new type of hot-rolled multiphase medium-Mn steels subjected to a multi-step quenching and partitioning heat treatment.

The present study was limited to two plate thicknesses, one strain rate (0.001 s^−1^), one quenching temperature (240 °C), and one partitioning temperature and time (450 °C, 300 s). Future studies should further explore these parameters to understand the impact of plate thicknesses and specimen geometry on mechanical properties. Furthermore, combining multilinear modeling with advanced ductile fracture models should address the limitation of multilinear material modeling in capturing total deformation at fracture points.

## Figures and Tables

**Table 1:** Chemical composition of investigated steel.

Element	C	Mn	Al	Si	Nb	Fe
Weight %	0.17	5.0	0.76	0.88	0.063	Bal.

**Table 2:** True stresses and true strains corresponding to engineering values of YS, UTS, TEl, and UEl.

Variant	Specimen Type	$\mathrm{True}\;\mathrm{Yield}\;\mathrm{Stress},\;\sigma_{T\;YS0.2}$, (Mpa)	True Ultimate Tensile Strength, $\sigma_{T\;UTS}$, (Mpa)	Elastic Modulus, E (GPa)	Poisson Ratio, ν	True total Elongation, $\varepsilon_{T\;TEl}$	True Uniform Elongation, $\varepsilon_{T\;UEl}$
Q240P450(300 s)	Round-12 mm plate	684	1514	197	0.3	0.137	0.097
Standard deviation		±18	±16	±15	0.3	±0.004	±0.005
Q240P450(300 s)	Flat-7 mm plate	809	1423	236	0.3	0.103	0.074
Standard deviation		±38	±22	±17	0.3	±0.026	±0.010

**Table 3:** Comparison of the experimental and FEM results for true stresses and true strains.

Property	12 mm Plate (Round Specimen)			7 mm Plate (Flat Specimen)		
	Experimental	FEM	Error (%)	Experimental	FEM	Error (%)
$\mathrm{True}\;\mathrm{ultimate}\;\mathrm{tensile}\;\mathrm{strength},\;\sigma_{T\;UTS},$ [MPa]	1514	1514	0	1423	1496	5.1
$\mathrm{True}\;\mathrm{total}\;\mathrm{elongation},\;\varepsilon_{T\;TEl}$	0.137	-	-	0.103	-	-
$\mathrm{True}\;\mathrm{uniform}\;\mathrm{elongation},\;\varepsilon_{T\;UEl}$	0.097	0.109	12.3	0.074	0.083	12.1

## Data Availability

The original contributions presented in this study are included in the article. Further inquiries can be directed to the corresponding author.
